# Friendship chemistry: An examination of underlying factors^☆^

**DOI:** 10.1016/j.soscij.2015.01.005

**Published:** 2015-02-18

**Authors:** Kelly Campbell, Nicole Holderness, Matt Riggs

**Affiliations:** California State University, San Bernardino

**Keywords:** Factor analysis, friendship formation, interpersonal chemistry, relationship initiation

## Abstract

Interpersonal chemistry refers to a connection between two individuals that exists upon first meeting. The goal of the current study is to identify beliefs about the underlying components of friendship chemistry. Individuals respond to an online Friendship Chemistry Questionnaire containing items that are derived from interdependence theory and the friendship formation literature. Participants are randomly divided into two subsamples. A principal axis factor analysis with promax rotation is performed on subsample 1 and produces 5 factors: Reciprocal candor, mutual interest, personableness, similarity, and physical attraction. A confirmatory factor analysis is conducted using subsample 2 and provides support for the 5-factor model. Participants with agreeable, open, and conscientious personalities more commonly report experiencing friendship chemistry, as do those who are female, young, and European/white. Responses from participants who have never experienced chemistry are qualitatively analyzed. Limitations and directions for future research are discussed.

## 1. Introduction

Interpersonal chemistry is a relatively new concept and although no predominant definition exists, it is described as an instant emotional and psychological connection between two individuals (Ceccoli, 2004; Swann, Sellers, & McClarty, 2006). The few researchers who examine this construct focus on sexual, rather than friendship chemistry (Leiblum & Brezsnyak, 2006; Liebowitz, 1983). Given that a person is likely to partake in a greater number of friendships versus romantic relationships over a lifetime, a thorough exploration of the factors involved in friendship formation, such as chemistry, is essential to this body of work (Sprecher & Regan, 2002). In the present study, we use interdependency theory (Rusbult & Van Lange, 2003; Kelley & Thibaut, 1978) and the friendship formation literature to explore the core components of friendship chemistry.

Researchers propose that friendship formation is a process that occurs relatively quickly. For example, Berg and Clark (1986) speculate that during the initial moments of an interpersonal encounter, individuals are already making decisions about which relationship type--friend or acquaintance--to pursue. Similarly, Abelson (1976) suggests that scripts exist for different kinds of relationships and after meeting someone only once, it is evident which script the relationship will follow. Berg (1984) demonstrates that students' satisfaction with their roommate after 2 weeks and 6 months of acquaintance is equally predictive of their choice to live with that roommate in the future. These findings suggest that the decision to pursue a friendship is relatively stable and may be predicted from the earliest phases of meeting.

We speculate that friendship chemistry is driven by a combination of relationship formation factors. Lieblum and Breznyak (2006) theorize that “sexual, or romantic, chemistry may reflect an overall global assessment of the quality of the sexual relationship based on multiple factors” (Lieblum and Breznyak, p. 56). In other words, sexual chemistry is likely to emerge from an interaction of the various elements that elicit romantic relations. Ambady, Bernieri, and Richeson (2000) indicate that people make decisions about whether to pursue a romantic or companionate relationship within moments of first meeting. Consequently, we propose that friendship chemistry results from an interaction of the most salient friendship formation characteristics within an initial interaction.

In order to determine the most relevant elements of rapid friendship formation, all factors should be assessed in a single study (Fehr, 2008). Unfortunately, a comprehensive list of factors has not been produced. Aron and colleagues (1989) examine the process of Falling-in-Friendship (FIF). Their study provides a list of factors that facilitate friendship development but does not focus on an initial interaction. Sprecher (1998) compiles and assesses 14 variables associated with friendship formation but omits factors such as sense of humor (Fehr, 2008; Sprecher & Regan, 2002) and communication (Sprecher & Duck, 1994). Knapp and Harwood (1977) similarly examine 39 characteristics associated with friendship formation and do not assess sense of humor. Given that these variables are not collectively examined, it remains difficult to determine the most salient factors involved in friendship formation.

## 2. Literature overview

Interdependence theory helps explain why the convergence of relevant friendship formation factors would result in chemistry. The theory states that individuals are dependent on relational partners for need fulfillment or rewarding outcomes; thus, relationship formation is based on a rewards/costs analysis in which rewards refer to the benefits acquired through pleasurable experiences and costs pertain to expenditures that result from unsatisfying ones (Kelley & Thibaut, 1978; Rusbult & Van Lange, 2003). A profitable relationship results when the rewards associated with a relationship outweigh the costs. Whether a relationship's outcome will be positive or negative is contingent on the ratio of rewards to costs and the availability of a more profitable alternative. If an individual perceives a relationship to be rewarding and does not foresee better alternatives, they will depend on their partner for rewarding outcomes and seek to maintain the connection. For example, Jane may rely on Mary for social support, because there is no one else to turn to. Mary, however, may have plenty of options for social support, but rely on Jane for help with schoolwork. Even though Jane and Mary provide different benefits to one another, the relationship is mutually rewarding, and therefore, a state of interdependence exists.

Many empirically supported friendship formation factors can be understood in terms of interdependence theory. One of the most widely recognized factors is similarity (Rivas, 2009; Sprecher, 2014). Similar behaviors and attitudes among individuals create “coordination” in a relationship and are “symmetrically facilitative,” whereas dissimilar behaviors and attitudes are “symmetrically interfering” (Kelley & Thibaut, 1978, pp. 66-67). Therefore, people are likely to find more enjoyment—and consequently more rewards—from relationships that are in sync versus discordant. Those with comparable demographic traits, intelligence, personality traits, attitudes, beliefs, and hobbies are more likely to form friendships with each other than people who are not similar on at least one or some combination of these dimensions (Fehr, 2008; Perry, 2013b). Interestingly, similarity of physical attractiveness also affects friendship formation. Cash and Derlega (1978) ask judges to rate pictures of male and female same-sexed friends and find that pictures of actual friendship pairs are rated as more similar in attractiveness than artificial pairs. People are drawn to physically attractive individuals because such individuals are assumed to possess desirable qualities such as social and professional happiness and a high occupational status (Dion, Berscheid, & Walster, 1972; Feingold, 1992).

Communication and mutual self-disclosures are relevant to friendship formation. Sprecher and Duck (1994) find that the quality of communication between two people significantly influences each person's desire for friendship with the other. People who communicate in a “personal, smooth, efficient, important, and satisfying” way are preferred over those who do not converse in such a manner (Sprecher and Duck, p. 3). Sprecher and Regan's (2002) research further reveals that expressive and open communication is highly valued across all relationship types including friendships. A related communication construct, self-disclosure, facilitates friendships (Clark et al., 2004; Sprecher et al., 2013). Archer, Berg, and Runge (1980) find that college students who reciprocally disclose highly intimate information to each other, such as their experiences of falling in love, report greater closeness than student participants who disclose more superficial information, such as sharing things they like about their classes. Greater numbers of disclosures as well as more intimate disclosures increase interpersonal closeness, and thereby, facilitate friendship formation.

Reciprocal liking, personableness, and sense of humor influence friendship formation. Beckman and Secord (1959) perform one of the earliest studies investigating the effects of reciprocal liking on groups of same-sex participants. Before the first group meeting, researchers tell participants that they can predict which individuals in the group will like them. The predictions are arbitrary, yet participants indicate a stronger liking for those who are expected to respond favorably toward them. Sprecher (1998) finds that reciprocal liking is a significant determinant of interpersonal attraction across romantic relationships, same-sex friendships, and opposite-sex friendships. Personableness, or the expression of warmth, kindness, consideration, and understanding, is shown to elicit interpersonal attraction for both genders across relationship types (Knapp & Harwood, 1977; Sprecher, 1998; Sprecher & Regan, 2002). Regarding sense of humor, Fraley and Aron (2004) randomly divide participants into same-sex pairs and ask them to either perform humorous tasks or non-humorous tasks together. Participants are more likely to report feeling interpersonal attraction to their partner if they share a humorous interaction. An individual's sense of humor mediates the association between sharing a humorous experience and feelings of closeness. Therefore, sharing humorous experiences and having a good sense of humor are important in the friendship formation process. Teger, Sprecher, and Erber (2013) find similar results in their recent study with college students.

Multiple studies show that situational factors impact friendship formation such as when people expect to interact with someone in the future (Fehr, 2008). In these cases, individuals tend to emphasize their partner's favorable qualities, while disregarding undesirable ones, so as to ensure that future encounters are enjoyable. Segal (1974) examines friendships formed between police trainees whose seats in a classroom are alphabetized. Friendships are most likely to occur between trainees whose last names are alphabetically similar, and therefore, seated near each other. More recently, Perry (2013a) finds that interracial friendships are more likely to form when individuals share a workplace or neighborhood. Chen et al. (2013) also examines the workplace and finds that fair, harmonious manager-employee relationships facilitate friendships among coworkers. Consequently, a friendship might form not because interactions are particularly rewarding, but out of convenience or because future interactions are inevitable. Although contextual factors provide opportunities for friendship formation, they do not explain the strong connection that exists when two people first meet.

The literature reviewed thus far indicates that various individual and dyadic factors are involved in friendship formation. The individual factors include attractiveness, communication skill, personableness, and sense of humor. The dyadic factors are similarity, mutual self-disclosure, and reciprocal liking. In the current study, we collectively explore individual and dyadic factors that influence participants' beliefs about friendship chemistry. Two sets of analyses are completed to investigate the core components of this construct. First, an exploratory analysis is used to identify factors that participants consider most important for friendship chemistry. Next, a confirmatory analysis is used to test whether the emergent factors are supported with different individuals.

## 3. Methodology

### 3.1. Sampling and participants

Prior to conducting our analyses, we first remove individuals from the dataset who have never experienced friendship chemistry (n = 42). Participants for the exploratory and confirmatory analyses are derived using 50% random split sampling. All participants reside in the United States and are recruited through professional listservs, websites (CraigsList.org), and university student participant pools. The first subsample (N = 688) is used for the exploratory factor analysis and consists of men (n = 81) and women (n = 607) ranging in age from 18 to 66 years (*Mean* = 24.84 years, *SD* = 8.34 years). A majority is residing in the Western U.S. (68%) and self-identify as European/white (43.5%) or Latino (37%) American. The second subsample (N = 715) is used for the confirmatory analysis and consists of men (n = 81) and women (n = 634) ranging in age from 18 to 65 years (*Mean* = 25.17 years, *SD* = 8.97 years). The majority is residing in the Western U.S. (64.1%) and self-identify as European/white (43.9%) or Latino (35.1%) American.

### 3.2. Procedure

The only requirement for study participation is that individuals be at least 18 years of age. After reading the online consent form and agreeing to participate in the study, they are presented with the following definition of friendship chemistry, “Friendship chemistry refers to an instant connection between friends that is easy and makes the relationship seem natural.” They are then asked whether they have ever experienced friendship chemistry. Participants who answer “yes” are asked to think of someone with whom they have experienced strong friendship chemistry and respond to a series of questions with that person in mind. Participants who respond “no” are asked an open-ended question about why they think they have not experienced it. Responses for both options are summarized in the results section. Participants also complete a personality assessment and demographics form. Upon finishing the survey, they have the option of entering a draw for a $50 gift card. University students also earn 2 extra credit points for their classes.

### 3.3. Measures

*Friendship chemistry* is assessed using the Friendship Chemistry Questionnaire (FCQ). The 35-item measure is developed for the present study using the empirical literature on friendship formation. The questionnaire consists of items to assess both individual and dyadic factors of friendship initiation. The individual factors assess attractiveness, communication, personableness, and sense of humor, and include items such as “I am sincere” and “My friend has a good sense of humor.” Dyadic factors assess similarity, mutual self-disclosure, and reciprocal liking and include items such as “I like my friend because he/she likes me” and “My friend and I share the same interests.” Responses are recorded on a 5-point Likert scale with options ranging from 1 (strongly disagree) to 5 (strongly agree). After performing an exploratory factor analysis on the 35-item scale (see below), the measure is modified to include a final set of 30 items. Cronbach's alpha coefficient for the 30-item FCQ is .93.

*Personality* is assessed using the International Personality Item Pool (IPIP; Goldberg, 1999). This is a 50-item scale that assesses the “Big Five” traits of extroversion, agreeableness, openness, emotional stability, and conscientiousness. Participants read a list of 50 statements (10 items per dimension) and indicate how much each statement applies to their personality using a 5-point Likert scale, with options ranging from 1 (very inaccurate) to 5 (very accurate). Cronbach's alpha coefficients in the present study are .87 for extroversion, .77 for agreeableness, .79 for openness, .86 for emotional stability, and .79 for conscientiousness.

*Demographic* data is collected for participants' sex, age, ethnicity, and region of residence within the U.S.

## 4. Results

### 4.1. Exploratory factor analyses

The 35 friendship chemistry items are analyzed using a principal axis factor analysis with promax rotation. The analysis reveals six factors, but the sixth factor consists only of items with higher loadings on other factors, suggesting that a 5-factor model is optimal. Five items are omitted due to low communalities of less than .200. Therefore, the analysis is conducted again using the 30 remaining items and forced to five factors. The five subscales account for 55.9% of the variance in friendship chemistry and are named: Reciprocal candor (α = .91), mutual interest (α = .87), personableness (α = .86), similarity (α = .74), and physical attraction (α = .91). Factor loadings for each subscale are shown in Table 1 and the correlations among the subscales are shown in Table 2.

### 4.2. Confirmatory factor analysis

A confirmatory factor analysis with maximum likelihood estimation is conducted using EQS 6 to test the hypothesized 5-factor model for friendship chemistry based upon the results of the exploratory factor analysis. The five proposed latent constructs (first order factors) include reciprocal candor, mutual interest, personableness, similarity, and physical attraction. The model also includes a sixth, second-order factor to represent overall friendship chemistry. The assumption of multivariate normality is violated; therefore, robust maximum likelihood estimation is used. The Satorra-Bentler Scaled *x*^2^, robust Comparative Fit Index (CFI), robust Root Mean-Square Error of Approximation (RMSEA), and normed chi-square test (chi-square divided by degrees of freedom) are used to interpret model fit. A CFI value greater than or equal to .90, RMSEA value less than .05, and normed chi-square value close to or less than 2 indicate a model of favorable fit (Hatcher, 2004; Kline, 2005).

**Figure 1:**
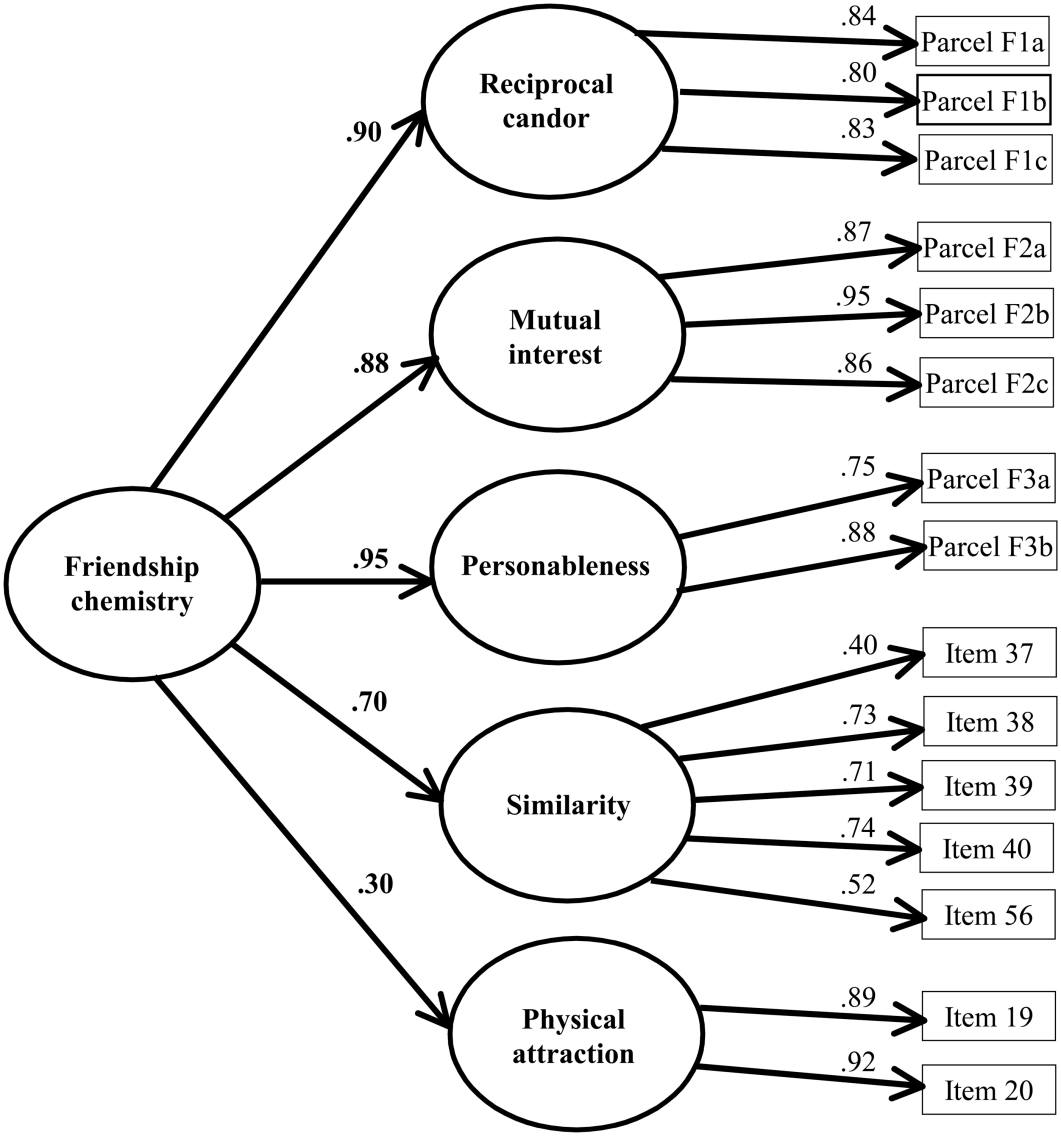
Confirmatory factor analysis model showing standardized robust maximum likelihood parameter estimates. Subscales consisting of 6 or more items were randomly parceled.

Based on the Lagrange Multiplier Test, three error covariances are allowed to relax the model and the Wald Test indicate that none of the measurement parameters need to be dropped. All fit indices reveal that the hypothesized 5-factor model is a good fit for the data (*x*^2^ = 163.38, df = 82; CFI = .97; RMSEA = .04; normed *x*^2^ = 1.99). All tested path coefficients are statistically significant. Moreover, a majority of the paths have coefficients above .70 with the first and second order factors. Modest path coefficients, ranging from .30 to .52 exist from friendship chemistry to physical attraction and from the similarity construct to items 37 (My friend and I have a similar level of education) and 56 (My friend and I have the same life goals). The 5-factor model with standardized path coefficients is shown in Figure 1.

### 4.3. Individual differences

In order to examine whether friendship chemistry differs based on personality or demographic traits, we conduct two regression analyses using the second sample. First, a linear regression analysis is performed to examine the association between friendship chemistry (summed score) and the Big Five personality traits. The model is significant (Adjusted R^2^ = .099, *p* < .001) and reveals that agreeableness (β = .179, *p* < .001), openness (β = .121, *p* < .001), and conscientiousness (β = .121, *p* < .001) are positively associated with friendship chemistry. A second linear regression is performed to examine the association between friendship chemistry and the demographic characteristics of sex, age, and ethnicity. The ethnic classifications are dummy coded into 0′s and 1′s. The model is significant (Adjusted R^2^ = .027, *p* < .001) and reveals that friendship chemistry is more common for individuals who are female (β = .102, *p* < .001), young (β = -.090, *p* < .001), and European/white (β = .141, *p* < .001).

We also examine qualitative responses for individuals who indicate that they have not experienced friendship chemistry (n = 42). The data are analyzed using the constant comparative method (Glaser & Strauss, 1967), which involves reading through responses and open coding the data for core themes. These themes are reflected here in italics. A majority of participants (37%) indicate being unsure about why they have not experienced chemistry (“I really don't know”). Several participants (24%) do not respond to the question, other than to state that they have not experienced it. A similar number (20%) believe that chemistry only exists between romantic partners (“Well when I experience it, I tend to have sexual relations with the person and then they are no longer a friend”). Some participants (9%) indicate that relationships take time to develop, or that friendship formation is not immediate (“Relationships are not connections that happen instantly. I'm more skeptical when meeting people”). A smaller number (5%) describe not having the opportunity to foster friendship chemistry (“I have never had much of a chance to make friends, or get close to anyone outside of family”). The same fraction of participants (5%) indicates that they have not met people with common interests or similarities (“People are not similar to me”) with whom to foster this type of connection.

## 5. Discussion

The goal of this study is to collectively explore individual and dyadic friendship formation factors to assess those most relevant to friendship chemistry. Five subscales emerge in the exploratory analysis: Reciprocal candor, mutual interest, personableness, similarity, and physical attraction. A confirmatory factor analysis reveals that the 5-factor model is a favorable fit. These results are consistent with our prediction that friendship chemistry is likely to occur when the most salient friendship formation factors converge and are balanced between individuals.

Although numerous friendship characteristics are assessed, only five factors are produced, which demonstrates the importance of simultaneously examining all variables together. Our collective assessment helps provide a concise and accurate conceptualization of the underlying dimensions of friendship chemistry. The factor of reciprocal candor contains items related to communication and self-disclosure; mutual interest pertains to having similar interests and humor; personableness reflects reciprocal liking and kindness/sincerity; similarity pertains to shared values and aspirations; and physical attraction contains items reflecting mutual attraction. Reciprocal candor and personableness are labeled using Knapp and Harwood's (1977) descriptors because these factors contain similar items in both studies.

Interestingly, although similarity emerges as a unique factor, four similarity items are omitted due to low communalities. According to Fabrigar and colleagues (1999), low communalities are likely to result when items are unreliable or unrelated to the main construct. Given that all omitted similarity items represent the construct of status homophily (similarity based on ascribed characteristics such as age, ethnicity, and income), we hypothesize that the low communalities likely occur because status homophily is not related to friendship chemistry. The similarity items that remain in the analysis are items that assess value homophily, which refers to the similarity of attitudes, beliefs, and aspirations. Fehr's (2008) review of studies that examine similarity between friends indicates that status and value homophily are both relevant to friendship formation; however, the results of our study suggest that only value homophily is relevant to friendship chemistry.

Given the combination of factors that result in friendship chemistry, it makes sense that characteristics such as similarity of age and ethnicity are not particularly relevant. Characteristics such as personableness and/or espousing a good sense of humor exist across age and ethnic groups, and would make individuals likely to experience chemistry, irrespective of their demographic differences. By contrast, if two individuals differ in regard to values or morals, other salient friendship chemistry factors might be affected. For example, if individuals do not respect each other's religious or cultural background, they would interact in a less personable and more unrewarding fashion, which would inhibit friendship chemistry.

The final item that is omitted after our exploratory analysis includes, “My friend has a social, extroverted personality.” Again, we hypothesize that the low communality of this item results from a lack of relation to the construct of friendship chemistry, not because the item is unreliable. Support for this hypothesis exists in our finding that extroversion is not related to friendship chemistry in the examination of personality traits.

All path coefficients in the confirmatory analysis are statistically significant but not all coefficients carry equal practical significance. The strength of the relationship between friendship chemistry and physical attraction is relatively weak in comparison to the other subscales, suggesting that physical attraction is less relevant to friendship chemistry. However, the physical attraction subscale only consists of two items, and some researchers suggest that at least three items are necessary to demonstrate a subscale's true reliability (Fabrigar et. al., 1999; Little, Lindenberger, & Nesselroade, 1999; Velicer & Fava, 1998). More items should be generated for the physical attraction subscale before its relevance to friendship chemistry can be confidently evaluated in future work.

The strength of the path coefficients from items 37 (My friend and I have a similar level of education) and 56 (My friend and I have the same life goals) to the similarity construct are also relatively weak. The low pathway coefficient for item 37 could occur for two reasons. As noted earlier, similarity of ascribed characteristics appears unrelated to the experience of friendship chemistry. According to McPherson, Smith-Lovin, and Cook (2001), education level is an ascribed status, and therefore, might account for the low path coefficient. However, this does not explain why education level remains in the initial analysis, whereas other status homophily items are omitted. Aside from being an ascribed status, acquired education might be considered an aspiration in that the education level an individual pursues is a personal decision. In this context, education level would be more consistent with the concept of value homophily, not status homophily, and could explain why it remained in the initial analysis. Similarly, item 56 (My friend and I have the same life goals) also describes a person's aspirations. Therefore, the low pathway coefficients of items 37 and 56 might indicate that shared aspirations are not as relevant to the similarity subscale compared to other items that assess shared values, beliefs, and morals.

The personality analysis reveals that agreeable, open, and conscientious traits are associated with friendship chemistry. Items on the agreeable and openness subscales correspond with items on the FCQ, so these positive associations are to be expected. For example, agreeableness is assessed with items such as “I have a soft heart and I am not interested in other people's problems” (reversed), which can be equated with the personableness items on the FCQ (“I am a warm and caring person”, “I care about the general well-being of others”). Similarly, openness is assessed with items such as “I am quick to understand things” and “I spend time reflecting on things”, which might compare to communication items on the FCQ (“The communication between my friend is easy and effortless”, “I feel like my friend really understands me”). Conscientiousness may associate with friendship chemistry due to the nature of our sample. Our participants are largely recruited from professional and university sources, making them more likely to espouse conscientious traits (“I follow a schedule and I am exacting in my work”). Given that students and professionals exhibit these qualities, they would likely find it rewarding to meet other people who are conscientious. Finally, all three personality traits are associated with good communication skills (McCrae & John, 1992). Given that the factors in the current study highlight the importance of communication, it follows that agreeable, open, and/or conscientious characteristics would facilitate friendship chemistry.

The demographic analyses reveal that women, younger participants, and those with a European/white ethnic background may experience friendship chemistry more than individuals from the other groups. With respect to gender, women and men receive differential socialization about relationships and communication. Given that some of the core elements of friendship chemistry relate to self-disclosure and communication, characteristics that are more encouraged among women, they may foster stronger connections in a first interaction compared to men. It is important to note however, that our sample contains more women than men, so these gender differences should be interpreted with caution. Future work might focus on the extent to which men and women differentially experience friendship chemistry.

Regarding age, it is expected that friendship chemistry might decrease with age due to family and work demands, which would limit the opportunity and energy for friendship formation. Older individuals are also more likely to be involved in a marriage or cohabiting union, which may encourage couple, rather than individual-based friendships. This assertion is supported by prior work indicating that individuals evaluate their existing relationships when deciding whether to form new relationships, and establish new connections only when there is reason to do so (Rusbult & Van Lange, 2003).

With respect to ethnicity, one possibility for our finding that friendship chemistry is more commonly reported among European/white participants is that compared to ethnic minority individuals, they are less likely to experience or think about racial discrimination (Marger, 2011). As such, they may be less cautious in their initial interactions, which would optimize their chances of friendship chemistry. In light of these findings, researchers should continue to evaluate the construct of friendship chemistry, including whether items on the FCQ accurately capture its underlying dimensions for people of diverse backgrounds.

## 6. Limitations and future research

A possible limitation of this research is that friendship means different things to different people (Selfhout et al., 2009). Sunnafrank and Ramirez (2004) suggest that although people tend to form friendships because of the rewards associated with interpersonal relations, rewards are subjectively evaluated. In other words, the factors that elicit friendship chemistry may vary depending on the population from which the sample is drawn. The current study uses U.S. samples, which consist of mostly European/white and Latino individuals. Therefore, the results may not generalize to participants of other ethnicities, or participants living outside of the U.S. However, few researchers examine friendship formation among ethnic minorities, and the large number of Latinos in our study extends prior work.

Our samples also contain many young adults and an examination of friendship chemistry in predominantly middle-aged or older samples might yield different findings. As previously noted, individuals become busy with career and family obligations when they get older, and may have less time and energy for friendship formation. Researchers should therefore continue to examine this construct with individuals at various stages of the lifespan such as childhood, adolescence, adulthood, and older adulthood, and in varying relationship statuses including single, married, and divorced.

Another limiting factor of the current study is that we do not require participants to specify whether they are thinking about a same-sex friend or opposite-sex friend when responding to the FCQ. The literature indicates that differences may exist in trait preferences between same-sex and opposite-sex friends. For example, Sprecher and Regan (2002) survey individuals about their romantic relationships, opposite sex-friendships, and same-sex friendships and find that physical attraction is most strongly associated with romantic partnerships, intermediately associated with opposite-sex friendships, and least associated with same-sex friendships. Additionally, compared to romantic partners and opposite-sex friendships, same sex-friendships are more likely to be based on similar attitudes and values. Gender differences regarding friendship preferences may also exist. For instance, Lewis et al. (2011) find that men prioritize physical attractiveness more than women in opposite-sex friendships. These findings suggest that the salience of traits varies depending on the relationship type. Future work should focus on pairs of same-sex and opposite-sex friends in order to expand upon the current study's findings.

Future research could also benefit from adding items to the FCQ that assess the rapid connection component of chemistry. Although friendship chemistry is defined in the present study by an instant connection, which is provided to participants, it is only assessed with one item on the scale (“My friend and I had an instant connection”). This item loads onto reciprocal candor and might indicate that reciprocal candor is most salient to the connection that individuals experience. It is noteworthy though, that compared to other items on the reciprocal candor factor, instant connection has the lowest factor loading. This may suggest that a separate factor for instant connection would emerge if assessed with multiple items. In order to disentangle the elements that elicit a rapid connection, future research should include additional items for this attribute in the FCQ. The inclusion of multiple items would help distinguish between qualities that lead to friendship and factors that measure the instant connection component of chemistry.

A final limitation of our study is its retrospective design. Participants may be influenced by their present day friendship and provide a biased description of their first encounter. For example, they might describe their friend as having a good sense of humor from the beginning when, in actuality, the friend's sense of humor does not become evident until later in the relationship. Similarly, studies have shown that beliefs about an occurrence do not always coincide with the actual experience (Wilson & Dunn, 2004). Researchers can overcome this issue in future work by implementing a “speed friending” design, in which participants are assessed immediately after meeting, and then followed over time to examine whether lasting friendships develop.

## 7. Conclusion

To the best of our knowledge, our study is the first to comprehensively examine beliefs about friendship chemistry. Understanding processes relevant to friendship formation, such as chemistry, or the specific factors involved in an initial interaction that lead to a relationship is important. Research shows that physical health, mental health, and overall life satisfaction are affected by a person's ability—or inability—to experience successful interpersonal relations (Leone & Hawkins, 2006). Responses from our qualitative data indicate that feelings of loneliness and dissatisfaction may emerge when a person has not experienced friendship chemistry. Understanding the individual and dyadic characteristics that lead to relationship formation helps researchers and clinicians move one step closer to enhancing the lives of those who struggle with this process.

## Figures and Tables

**Table 1:** Friendship chemistry: Subscales, items, and factor loadings for exploratory factor analysis with promax rotation

*Reciprocal candor*	
I feel like my friend really understands me.	.98
I feel I can tell my friend anything.	.85
I feel like I really understand my friend.	.84
My friend feels like he/she can tell me anything.	.80
The communication between my friend and I is easy and effortless.	.70
My friend feels that he/she can trust me.	.70
I feel like I can trust my friend.	.67
My friend and I had an instant connection.	.51
*Mutual interest*	
My friend finds me funny.	.87
I find my friend funny.	.86
I find my friend interesting.	.65
My friend and I find the same things funny.	.64
I feel good when I am around my friend.	.59
My friend finds me interesting.	.55
I get excited to talk to or see my friend.	.44
My friend and I share the same interests.	.39
*Personableness*	
I care about the general well-being of other people.	.81
I am a warm and caring person.	.80
I am a down-to-earth, genuine person.	.67
My friend is a warm and caring person.	.57
My friend cares about the general well-being of other people.	.49
My friend is a down-to-earth, genuine person.	.46
I like my friend because he/she likes me.	.25
*Similarity*	
My friend and I have similar values.	.92
My friend and I have similar morals.	.92
My friend and I have similar beliefs about life.	.66
My friend and I have the same life goals.	.34
My friend and I have a similar level of education.	.32
*Physical attraction*	
I find my friend physically attractive.	.91
My friend finds me physically attractive.	.90

Source: Author calculations using EQS.

Note. Six items were dropped because they loaded onto more than one factor.

**Table 2:** Factor correlations for friendship chemistry subscales

	Reciprocal candor	Mutual interest	Personableness	Similarity	Physical attraction
Reciprocal candor	1.00	.601	.741	.480	.278
Mutual Interest		1.00	.449	.578	.225
Personableness			1.00	.404	.314
Similarity				1.00	.196
Physical Attraction					1.00

Source: Author calculations using SPSS.

Notes: Intercorrelations among tabulated subscales from subsample 1.

## Highlights

- The underlying factors of friendship chemistry are: reciprocal candor, mutual interests, personableness, similarity, and physical attraction.
- People with agreeable, open, and conscientious personalities more commonly report friendship chemistry.
- Women, young, and European/white participants more commonly report friendship chemistry.
- Reasons for not experiencing friendship chemistry include believing that chemistry only happens in romantic relationships, believing that friendships take time to develop, reduced opportunities for fostering chemistry, and not having met people with similar interests.

## References

[R1] Abelson RP, Carrol J, Payne J (1976). Script processing in attitude formation and decision making. Cognition and social behavior.

[R2] Ambady N, Bernieri FJ, Richeson JA, Zanna MP (2000). Toward a histology of social behavior: Judgmental accuracy from thin slices of the behavioral stream. Advances in experimental social psychology.

[R3] Archer RL, Berg JH, Runge TE (1980). Active and passive observers' attraction to a self-disclosing other. Journal of Experimental Social Psychology.

[R4] Aron A, Dutton DG, Aron EN, Iverson A (1989). Experiences of falling in love. Journal Of Social And Personal Relationships.

[R5] Beckman CW, Secord PF (1959). The effect of perceived liking on interpersonal attraction. Human Relations.

[R6] Berg JH (1984). The development of friendship between roommates. Journal of Personality and Social Psychology.

[R7] Berg JH, Clark MS, Derlega V, Winstead B (1986). Differences in social exchange between intimate and other relationships: Gradually evolving or quickly apparent?. Friendship and social interaction.

[R8] Cash TF, Derlega VJ (1978). The matching hypothesis: Physical attractiveness among same-sexed friends. Personality and Social Psychology Bulletin.

[R9] Ceccoli VC (2004). Finding home in (an)other: Relational chemistry and its psychoanalytic derivations. Psychoanalytic Dialogues.

[R10] Chen C, Mao HY, Hsieh AT, Liu L, Yen C (2013). The relationship among interactive justice, leader–member exchange, and workplace friendship. Social Science Journal.

[R11] Clark RA, Dockum M, Hazeu H, Huang M, Luo N, Ramsy J, Spyrou A (2004). Initial encounters of young men and women: Impressions and disclosure estimates. Sex Roles.

[R12] Dion K, Berscheid E, Walster E (1972). What is beautiful is good. Journal of Personality and Social Psychology.

[R13] Fabrigar LR, Wegener DT, MacCallum RC, Strahan EJ (1999). Evaluating the use of exploratory factor analysis in psychological research. Psychological Methods.

[R14] Fehr B, Sprecher S, Wenzel A, Harvey J (2008). Friendship formation. Handbook of relationship initiation.

[R15] Fraley B, Aron A (2004). The effect of a shared humorous experience on closeness in initial encounters. Personal Relationships.

[R16] Glaser B, Strauss AL (1967). The discovery of grounded theory: Strategies for qualitative research.

[R17] Goldberg LR, Mervielde I, Deary I, De Fruyt F, Ostendorf F (1999). A broad-bandwidth, public domain, personality inventory measuring the lower-level facets of several five-factor models. Personality Psychology in Europe.

[R18] Hatcher L (2004). A step-by-step approach system to using the SAS system for factor analyses and structural equation modeling.

[R19] Kelley HH, Thibaut JW (1978). Interpersonal relations: A theory of interdependence.

[R20] Kline RB (2005). Principles and practices of structural equation modeling.

[R21] Knapp CW, Harwood BT (1977). Factors in the determination of intimate same-sex friendships. Journal of Genetic Psychology.

[R22] Leiblum S, Brezsnyak M (2006). Sexual chemistry: Theoretical elaboration and clinical implications. Sexual and Relationship Therapy.

[R23] Leone C, Hawkins LB (2006). Self-monitoring and close relationships. Journal of Personality.

[R24] Lewis DG, Conroy-Beam D, Al-Shawaf L, Raja A, DeKay T, Buss DM (2011). Friends with benefits: The evolved psychology of same- and opposite-sex friendship. Evolutionary Psychology.

[R25] Liebowitz MR (1983). The chemistry of love.

[R26] Little TD, Lindenberger U, Nesselroade JR (1999). On selecting indicators for multivariate measurement and modeling with latent variables: When “good” indicators are bad and “bad” indicators are good. Psychological Methods.

[R27] Marger MN (2011). Race and Ethnic Relations: American and Global Perspectives.

[R28] McCrae RR, John OP (1992). An introduction to the five-factor model and its applications. Journal of Personality.

[R29] McPherson M, Smith-Lovin L, Cook M (2001). Birds of a feather: Homophily in social networks. Annual Review of Sociology.

[R30] Perry SL (2013a). Racial composition of social settings, interracial friendship, and whites' attitudes toward interracial marriage. Social Science Journal.

[R31] Perry SL (2013b). Are interracial daters more supportive of same-sex unions?. Social Science Journal.

[R32] Rivas J (2009). Friendship selection. International Journal of Game Theory.

[R33] Rusbult CE, Van Lange PAM (2003). Interdependence, interaction, and relationships. Annual Review of Psychology.

[R34] Segal MW (1974). Alphabet and attraction: An unobtrusive measure of the effect of propinquity in field setting. Journal of Personality and Social Psychology.

[R35] Selfhout S, Denissen J, Branje S, Meeus W (2009). In the eye of the beholder: Perceived, actual, and peer-rated similarity in personality, communication, and friendship intensity during the acquaintanceship process. Journal of Personality and Social Psychology.

[R36] Sprecher S (1998). Insiders' perspectives on reasons for attraction to a close other. Social Psychology Quarterly.

[R37] Sprecher S (2014). Effects of Actual (Manipulated) and Perceived Similarity on Liking in Get-acquainted Interactions: The Role of Communication. Communication Monographs.

[R38] Sprecher S, Duck S (1994). Sweet talk: The importance of perceived communication for romantic and friendship attraction experienced during a get-acquainted date. Personality and Social Psychology Bulletin.

[R39] Sprecher S, Regan PC (2002). Liking some things (in some people) more than others: Partner preferences in romantic relationships and friendships. Journal of Social and Personal Relationships.

[R40] Sprecher S, Treger S, Wondra JD, Hilaire N, Wallpe K (2013). Taking turns: Reciprocal self-disclosure promotes liking in initial interactions. Journal Of Experimental Social Psychology.

[R41] Sunnafrank M, Ramirez A (2004). At first sight: Persistent relational effects of get-acquainted conversations. Journal of Social and Personal Relations.

[R42] Swann WB, Sellers JG, McClarty KL (2006). Tempting today, troubling tomorrow: The roots of precarious couple effect. Personality and Social Psychology Bulletin.

[R43] Velicer WF, Fava JL (1998). Effects of variable and subject sampling on factor pattern recovery. Psychological Methods.

[R44] Wilson TD, Dunn E (2004). Self-knowledge: Its limits, value, and potential for improvement. Annual Review of Psychology.

